# Using a kernel density estimation based classifier to predict species-specific microRNA precursors

**DOI:** 10.1186/1471-2105-9-S12-S2

**Published:** 2008-12-12

**Authors:** Darby Tien-Hao Chang, Chih-Ching Wang, Jian-Wei Chen

**Affiliations:** 1 Department of Electrical Engineering, National Cheng Kung University, Tainan, 70101, Taiwan, R.O.C.

## Abstract

**Background:**

MicroRNAs (miRNAs) are short non-coding RNA molecules participating in post-transcriptional regulation of gene expression. There have been many efforts to discover miRNA precursors (pre-miRNAs) over the years. Recently, *ab initio *approaches obtain more attention because that they can discover species-specific pre-miRNAs. Most *ab initio *approaches proposed novel features to characterize RNA molecules. However, there were fewer discussions on the associated classification mechanism in a miRNA predictor.

**Results:**

This study focuses on the classification algorithm for miRNA prediction. We develop a novel *ab initio *method, miR-KDE, in which most of the features are collected from previous works. The classification mechanism in miR-KDE is the relaxed variable kernel density estimator (RVKDE) that we have recently proposed. When compared to the famous support vector machine (SVM), RVKDE exploits more local information of the training dataset. MiR-KDE is evaluated using a training set consisted of only human pre-miRNAs to predict a benchmark collected from 40 species. The experimental results show that miR-KDE delivers favorable performance in predicting human pre-miRNAs and has advantages for pre-miRNAs from the genera taxonomically distant to humans.

**Conclusion:**

We use a novel classifier of which the characteristic of exploiting local information is particularly suitable to predict species-specific pre-miRNAs. This study also provides a comprehensive analysis from the view of classification mechanism. The good performance of miR-KDE encourages more efforts on the classification methodology as well as the feature extraction in miRNA prediction.

## Background

MicroRNAs are short RNAs (~20–22 nt) that can regulate target genes by binding to the mRNAs for cleavage or translational repression [1-3]. The discovery of miRNA shows that RNA is not only a carrier of gene information, but also a mediator of gene expression. The first studied miRNAs are *lin-4 *and *let-7*, which have been found during studies of genetic defects in early larval *Caenorhabditis elegans *[4,5]. To date, 6396 miRNAs have been identified [6]. The rapid growth results from the development of not only the experiment techniques but also the computational methods [7].

One of the most extensively developed computational methods for miRNA detection is the comparative approach. The most straightforward method is to align unknown RNA sequences to known pre-miRNAs through NCBI BlastN [8]. Advanced comparative approaches to discover pre-miRNAs strongly rely on sequence similarity [9] or on sequence profiles [10]. One drawback of homology search is the generation of many false positives (RNAs containing no mature miRNA predicted to be pre-miRNAs). Subsequently, cross-species evolutionary conservation has been widely used to eliminate these false positives [11-19]. Another well known method to identify novel pre-miRNAs is using conservation patterns based on a set of homology sequences [20-22].

Comparative approaches heavily rely on sequence similarity to known pre-miRNAs, and suffer lower sensitivity in detecting novel pre-miRNAs without known homology pre-miRNAs [22,23]. To overcome this problem, many *ab initio *algorithms, requiring no sequence or structure alignment, have recently been developed to detect complete new pre-miRNAs for which no close homology are known [24-28]. Brameier and Wiuf [29] proposed a motif-based *ab initio *method, miRPred, yielded 90% sensitivity and 99.1% specificity for human miRNAs. These *ab initio *methods are suitable to predict species-specific and non-conserved pre-miRNAs, which occupy the majority of undiscovered pre-miRNAs [18]. Other methods improved the miRNA prediction by first predicting some miRNA-related motifs such as the conserved 7-mers in 3'-UTRs [30] and Drosha processing sites [31].

Among these *ab initio *methods, Sewer *et al*. [24] used base pair frequencies and quantifying certain pre-miRNA structure elements as the characteristic features and detected 71% of pre-miRNAs with a low false positive rate of ~3% for virus. Triplet-SVM [25] used the frequencies of structure-sequence triplets as the characteristic features and yielded an overall accuracy of 90.9% for 11 species. BayesMiRfind [26] used sequence and structure features with comparative post-filtering and delivered >80% sensitivity and >90% specificity for *C. elegans *and *Mouse*. RNAmicro [27] introduced the thermodynamic properties with multiple sequence alignment and yielded >90% sensitivity and >99% specificity for *C. elegans *and *C. briggsae*. MiPred [28] used dinucleotide frequencies, six folding measures and five normalized folding quantities as the characteristic features and yielded an overall accuracy of 95.6% for 40 species.

With the development of *ab initio *approaches, the characteristic features for describing RNA molecules have been extensively studied in recent years. However, there were fewer discussions on the associated classification mechanism. Most *ab initio *approaches proposed novel characteristic features, but adopted an off-the-shelf machine learning tool. Furthermore, most of them incorporated with the same classifier, support vector machine (SVM), because of its prevailing success in diverse bioinformatics problems [32-34].

In this study, we focus on the classification methodology for pre-miRNAs prediction. A novel *ab initio *method, miR-KDE, for identifying pre-miRNAs from other hairpin sequences with similar stem-loop features (we call them pseudo hairpins) is developed. The feature set comprises several sequence and structure characteristics collected from previous works. We incorporate the relaxed variable kernel density estimator (RVKDE) [35] to classify RNA sequences based on the feature set. RVKDE is an instance-based classifier that exploits more local information from the dataset than SVM. An analysis based on the decision boundary of classifiers is conducted in this study to elaborate this characteristic of RVKDE. The performance of miR-KDE is evaluated using a training set consisted of only human pre-miRNAs to predict a benchmark collected from 40 species. Experimental results show that miR-KDE delivers favorable performance in predicting human pre-miRNAs and has advantages for pre-miRNAs from the genera taxonomically distant to humans.

## Results and discussion

### Experimental results on human pre-miRNAs

The performances of triplet-SVM, miPred and the present miR-KDE in predicting human pre-miRNAs are shown in Table 1. The %SE, %SP, %ACC, %Fm and %MCC of miR-KDE of five-fold cross-validation on the HU400 dataset are 90.5%, 97.5%, 94.0%, 93.8% and 88.2%, respectively. Most of the five measures are superior to triplet-SVM and miPred, except that miPred delivers a higher %SP. The comparison based on HU400 must be taken carefully, of course, because the parameters of alternative predictors are determined to maximize the performance for this dataset. Next, the three predictors are evaluated using HU400 to predict the HU216 dataset. The %SE, %SP, %ACC, %Fm and %MCC of miR-KDE are 88.9%, 92.6%, 90.7%, 90.6% and 81.5%. These results demonstrate the good performance of miR-KDE in identifying human pre-miRNAs from pseudo hairpins.

**Table 1:** Performances of triplet-SVM, miPred and miR-KDE in predicting human pre-miRNAs.

	**%SE**	**%SP**	**%ACC**	**%Fm**	**%MCC**
Five-fold cross-validation on HU400					
triplet-SVM	86.5%	91.5%	89.0%	88.7%	78.1%
miPred	87.5%	**98.0%**	92.8%	92.3%	86.0%
miR-KDE	**90.5%**	97.5%	**94.0%**	**93.8%**	**88.2%**
Using HU400 to predict HU216					
triplet-SVM	83.3%	86.1%	84.7%	84.5%	69.5%
miPred	88.0%	88.0%	88.0%	88.0%	75.9%
miR-KDE	**88.9%**	**92.6%**	**90.7%**	**90.6%**	**81.5%**

The best performance among each dataset is highlighted in bold.

### Experimental results on non-human pre-miRNAs

Table 2 extends the evaluation to the NH3350 dataset, which includes 1675 non-human pre-miRNAs from 39 species and 1675 human pseudo hairpins. The %SE, %SP, %ACC, %Fm and %MCC of miR-KDE are 95.8%, 93.5%, 94.7%, 94.7% and 89.3%. Most of these results are superior to triplet-SVM and miPred except that miPred delivers a higher %SE. We thus provide a sensitivity of miR-KDE under the condition of having the same specificity as miPred in the last row of Table 2.

**Table 2:** Performances of triplet-SVM, miPred and miR-KDE in predicting non-human pre-miRNAs.

	**%SE**	**%SP**	**%ACC**	**%Fm**	**%MCC**
triplet-SVM	91.5%	88.7%	90.1%	90.2%	80.2%
miPred	96.7%	90.4%	93.6%	93.7%	87.3%
miR-KDE	95.8%	**93.5%**	**94.7%**	**94.7%**	**89.3%**
with miPred's %SP	**97.4%**	90.4%	93.9%	94.1%	88.1%

The best performance among each dataset is highlighted in bold.

A further analysis is conducted to compare miPred and miR-KDE because of their comparable performance in Table 2. Table 3 shows the performance of miPred and miR-KDE for the NH3350 dataset in terms of genus. This experiment divides the NH3350 dataset into five sub-datasets based on genus, where each sub-dataset contains equal number of pre-miRNAs and pseudo hairpins. The 1675 pseudo hairpins are randomly assigned to each sub-dataset without replacement. Table 4 shows the size of these sub-datasets.

**Table 3:** Performances of miPred and miR-KDE for the NH3350 dataset in terms of genus.

	**%SE**	**%SP**	**%ACC**	**%Fm**	**%MCC**
Vertebrata					
miPred	95.3%	88.8%	92.1%	92.3%	84.3%
miR-KDE	93.4%	**92.8%**	**93.1%**	**93.2%**	**86.3%**
with miPred's %SP	**96.1%**	88.8%	92.5%	92.7%	85.2%
Arthropoda					
miPred	98.8%	89.0%	93.9%	94.2%	88.2%
miR-KDE	**100.0%**	**92.0%**	**96.0%**	**96.2%**	**92.3%**
Viridiplantae					
miPred	98.2%	93.6%	95.9%	96.0%	91.9%
miR-KDE	**98.4%**	**95.0%**	**96.7%**	**96.8%**	**93.4%**
Nematoda					
miPred	**97.2%**	90.4%	93.8%	94.0%	87.8%
miR-KDE	**97.2%**	**92.7%**	**94.9%**	**95.0%**	**89.9%**
Viruses					
miPred	97.2%	93.1%	95.1%	95.2%	90.4%
miR-KDE	94.4%	**97.2%**	**95.8%**	95.8%	91.7%
with miPred's %SP	**98.6%**	93.1%	**95.8%**	**95.9%**	**91.8%**
Overall					
miPred	97.3% ± 1.3%	91.0% ± 2.3%	94.1% ± 1.5%	94.3% ± 1.4%	88.5% ± 2.9%
miR-KDE	96.7% ± 2.7%	**93.9% **± 2.1%	**95.3% **± 1.4%	**95.4% **± 1.4%	**90.7% **± 2.8%
with miPred's %SP	**98.1% **± 1.5%	92.3% ± 2.2%	95.2% ± 1.6%	95.3% ± 1.6%	90.5% ± 3.3%

The best performance among each dataset is highlighted in bold.

**Table 4:** Summary of sub-datasets derived from the NH3350 dataset.

**Genus**	**Number of pre-miRNAs**^1^	**Number of pseudo hairpins**^2^
Vertebrata	824	824
Arthropoda	163	163
Viridiplantae	439	439
Nematoda	177	177
Viruses	72	72
Overall	1675	1675

^1^Each sub-dataset contains pre-miRNAs from the corresponding genus. ^2^All sub-datasets contain pseudo hairpins collected from human genome.

In this experiment, miR-KDE yields superior performance to miPred in terms of %SP, %ACC, %Fm and %MCC for all the genera. With respect to the %SE, miR-KDE performs better in *Arthropoda*, *Viridiplantae *and *Nematoda*, but worse in *Vertebrata *and *Viruses *than miPred. This is particularly of interest since *Vertebrata *is the closest genus taxonomically to humans, while *Viruses *is the most distant genus taxonomically to humans, among the five genera. One reasonable explanation is that viruses lack miRNA processing proteins such as Drosha, Dicer and RISC [36]. Viral miRNAs utilize such processing proteins from their hosts to regulate viral expression after infecting [37,38]. Thus, viral-encoded pre-miRNAs are likely to have very similar characteristics to those pre-miRNAs from the host (*i.e.*, human). As a result, the good performance of using human pre-miRNAs to predict *Arthropoda*, *Viridiplantae *and *Nematoda *ones indicates that miR-KDE is suitable for detecting species-specific pre-miRNAs.

### Contribution of the classification mechanism

We next investigate the effect of using RVKDE by separating two differences of miR-KDE to miPred: 1) introducing the four stem-loop features and 2) using RVKDE instead of SVM. Table 5 shows the performance of four possible predictors by individually enabling/disabling the two differences. The best %SE, %SP, %ACC, %Fm and %MCC in Table 5 are achieved by predictors with the four stem-loop features, regardless of the classification mechanism and the testing set. This observation indicates that the four stem-loop features are helpful in identifying pre-miRNAs. In another respect, SVM delivers better %SE, while RVKDE delivers better %SP, regardless of the feature set and the testing set. With respect to the three overall measures, RVKDE performs almost identically to SVM for the HU216 dataset, and has some advantages for the NH3350 dataset. This reveals that the advantage of miR-KDE for specific-species miRNA prediction in Table 3 benefits mainly from the classification mechanism.

**Table 5:** Comparison of miPred and miR-KDE in terms of the feature set and the classification mechanism.

	**Without the four stem-loop features**^1^					**With the four stem-loop features**^2^				
		
	%SE	%SP	%ACC	%Fm	%MCC	%SE	%SP	%ACC	%Fm	%MCC
HU216^3^										
SVM	88.0%	88.0%	88.0%	88.0%	75.9%	**90.7%**	90.7%	**90.7%**	**90.7%**	**81.5%**
RVKDE	85.2%	90.7%	88.0%	87.6%	76.0%	88.9%	**92.6%**	**90.7%**	90.6%	**81.5%**
NH3350^4^										
SVM	96.7%	90.4%	93.6%	93.7%	87.3%	**97.3%**	91.3%	94.3%	94.4%	88.7%
RVKDE	94.8%	93.4%	94.1%	94.1%	88.2%	95.8%	**93.5%**	**94.7%**	**94.7%**	**89.3%**

The best performance among each dataset is highlighted with bold font. ^1^Using the 29 features in miPred. ^2^Using the 33 features in miR-KDE, *i.e.*, the 29 features derived from miPred and the four stem-loop features. ^3^Using the HU400 dataset to predict the HU216 dataset. ^4^Using the HU400 dataset to predict the NH3350 dataset.

### Decision boundaries of SVM and RVKDE

**Figure 1:**
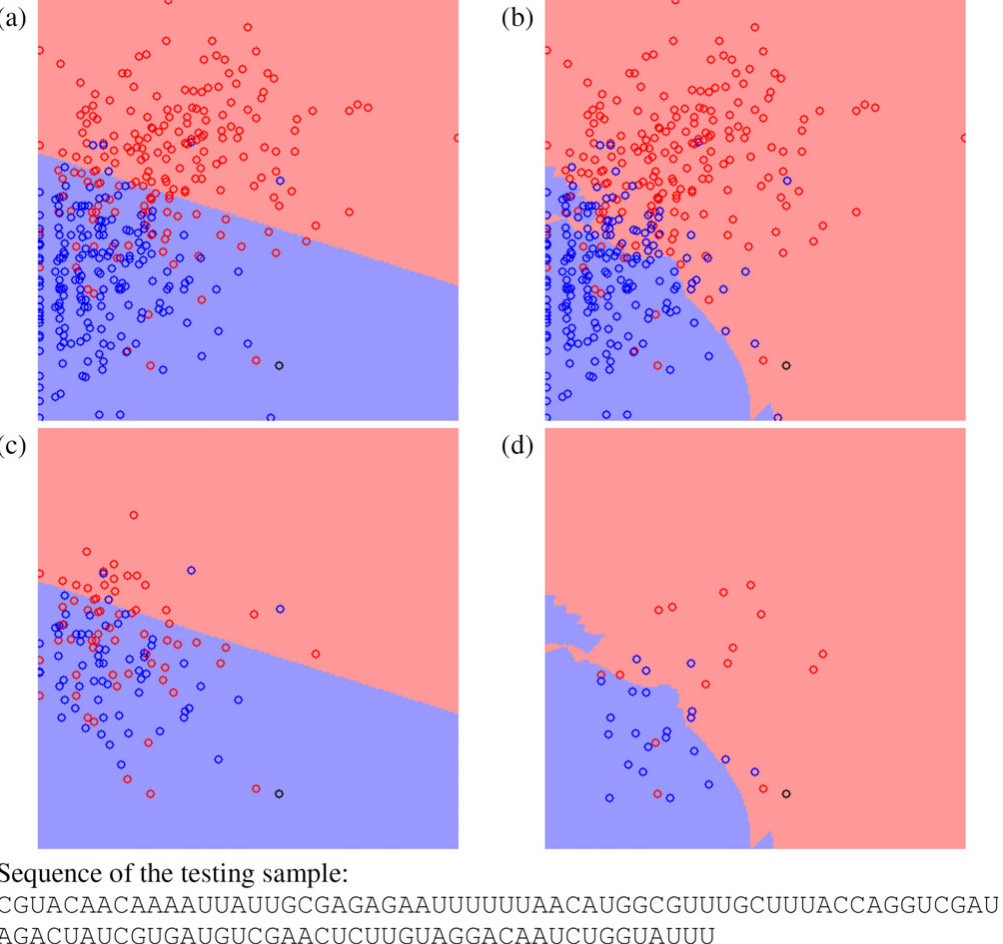
**The decision boundary plots, where (a) and (c) are generated by SVM and (b) and (d) are generated by RVKDE. **The *x*-axis is frequency of the dinucleotide "UU", and the *y*-axis is base pairing propensity[44]. The black circle is a testing pre-miRNA for the pre-miRNA *Caenorhabditis elegans *miR-260. The red and blue circles represent positive and negative training samples. In (c) and (d), training samples not involved in the decision function of the classifiers are removed.

To explain the characteristic of RVKDE in miRNA prediction, four cases are selected to demonstrate its difference to SVM from the view of decision boundary. For the four selected testing samples, miPred and miR-KDE make different predictions. In this analysis, miR-KDE adopts only 29 features derived from miPred to exclude the effect by introducing the four stem-loop features. Figure 1 shows a testing pre-miRNA, *Caenorhabditis elegans *miR-260, and the training samples from HU400 on the decision boundary plots. The black circle represents the testing sample, red circles represent the training pre-miRNAs and blue circles represent the training pseudo hairpins. The background color indicates the predictor's decision. The details of generating the decision boundary plots can be found in the 'Materials and methods' section.

In Figure 1(a) and 1(b), most the training samples locate at the top-left part in the plane. In this region, both SVM and RVKDE conclude that samples with larger *y*-axis tend to be pre-miRNAs and samples with smaller *y*-axis tend to be pseudo hairpins. The main inconsistence between the two classifiers occurs in the region including fewer training samples. Figure 1(c) and 1(d) hide the training samples that are not used to construct the decision boundary. Namely, Figure 1(c) shows only the support vectors, and Figure 1(d) shows only the *kt *nearest training samples to the testing sample (see the 'Materials and methods' section for details). In this example, RVKDE exploits more local information and generates an irregular decision boundary.

**Figure 2:**
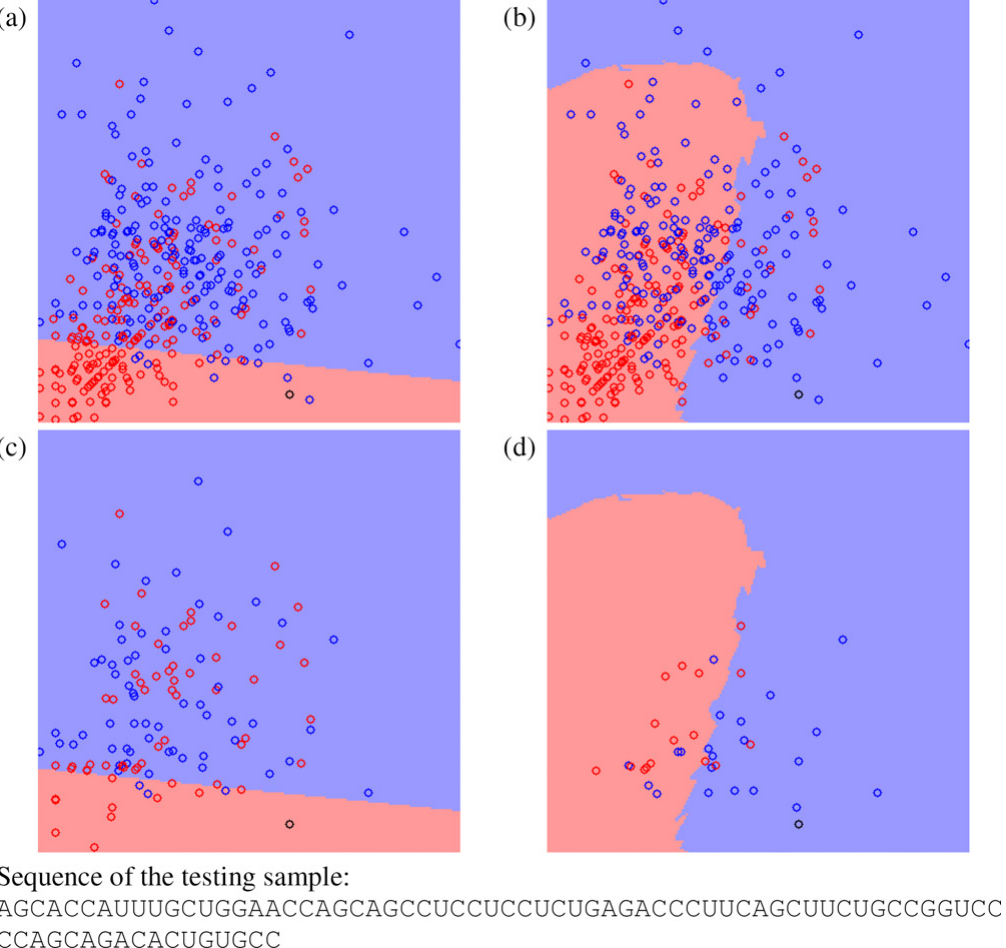
**The decision boundary plots, where (a) and (c) are generated by SVM and (b) and (d) are generated by RVKDE. **The *x*-axis is frequency of the dinucleotide "CC", and the *y*-axis is frequency of the dinucleotide "GG". The black circle is a testing pseudo hairpin. The red and blue circles represent positive and negative training samples. In (c) and (d), training samples not involved in the decision function of the classifiers are removed.

**Figure 3:**
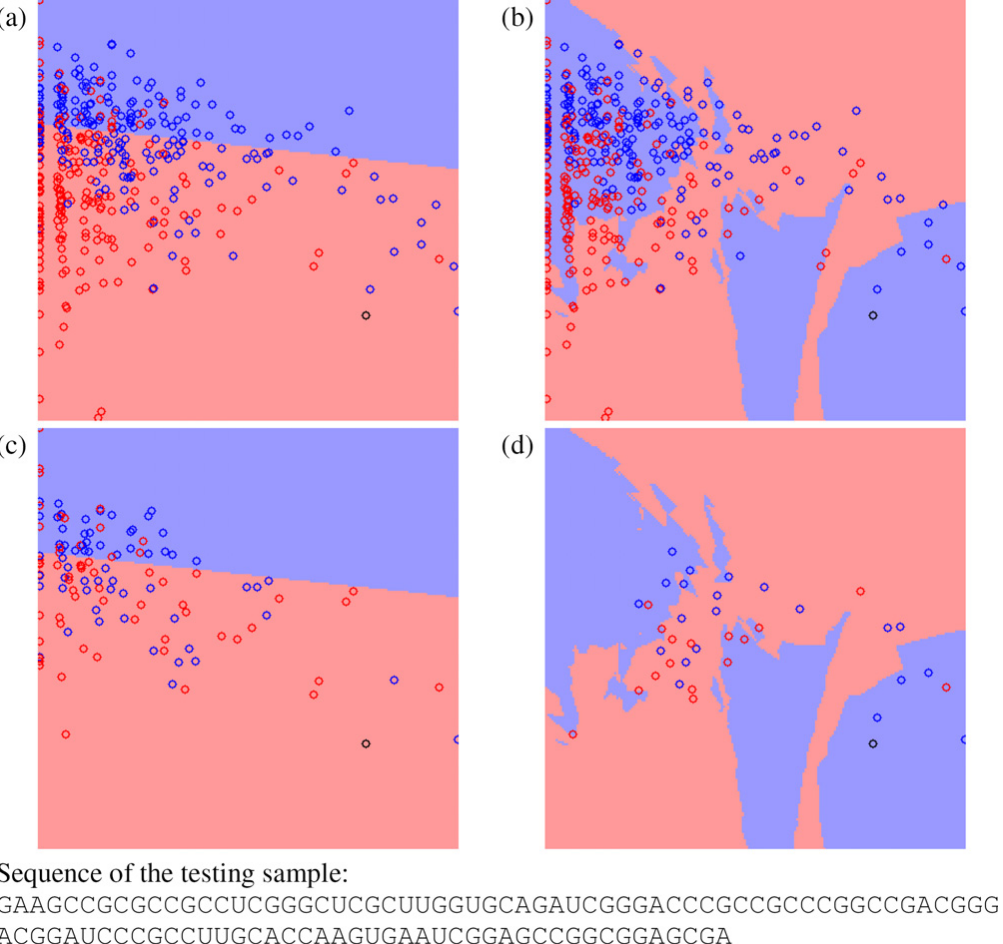
**The decision boundary plots, where (a) and (c) are generated by SVM and (b) and (d) are generated by RVKDE. **The *x*-axis is frequency of the dinucleotide "CG", and the *y*-axis is ratio of the minimum free energy to the sequence length[46]. The black circle is a testing pre-miRNA for the pre-miRNA *Zea mays *miR168a. The red and blue circles represent positive and negative training samples. In (c) and (d), training samples not involved in the decision function of the classifiers are removed.

**Figure 4:**
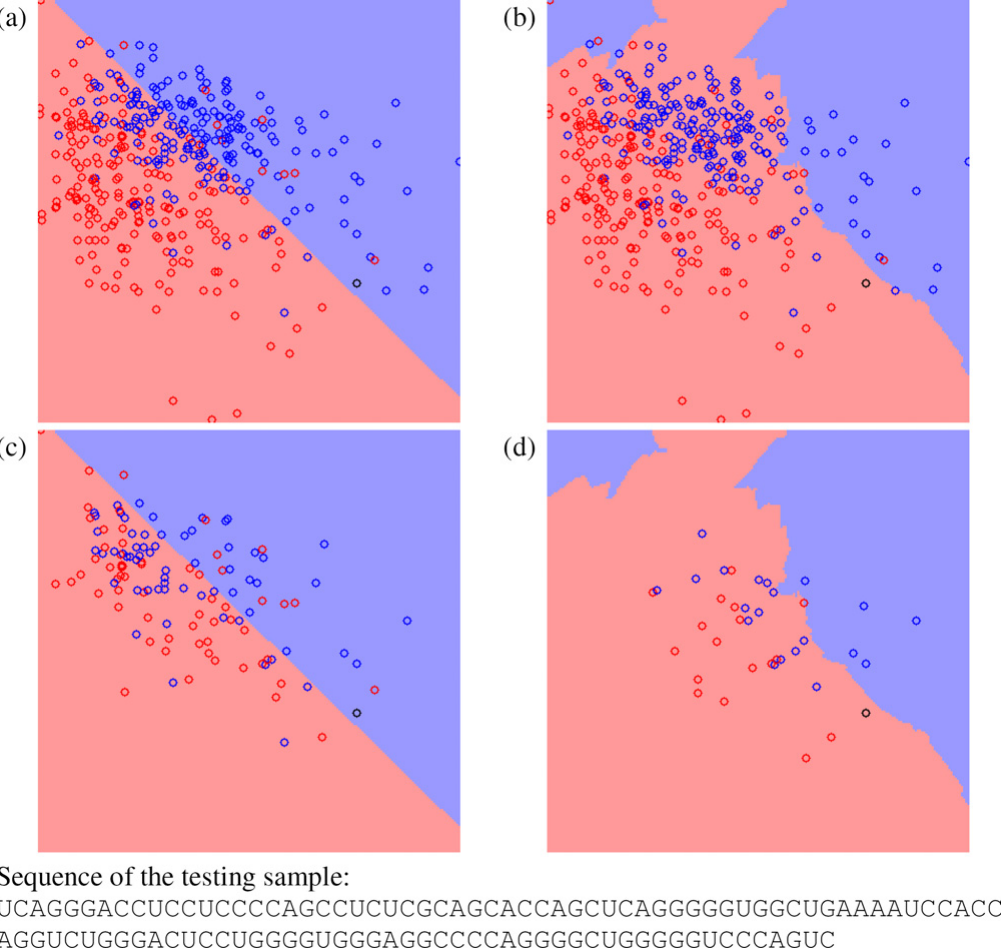
**The decision boundary plots, where (a) and (c) are generated by SVM and (b) and (d) are generated by RVKDE. **The *x*-axis is frequency of the dinucleotide "GG", and the *y*-axis is ratio of the minimum free energy to the sequence length[46]. The black circle is a testing pseudo hairpin. The red and blue circles represent positive and negative training samples. In (c) and (d), training samples not involved in the decision function of the classifiers are removed.

Figure 2, Figure 3 and Figure 4 show other three testing cases classified differently by miPred and miR-KDE. Figure 2 shows a pseudo hairpin classified incorrectly by miPred and correctly by miR-KDE. Figure 3 shows a pre-miRNA, *Zea mays *miR168a, classified correctly by miPred but incorrectly by miR-KDE. Finally, Figure 4 shows a pseudo hairpin correctly classified by miPred but incorrectly by miR-KDE. All these figures have a common characteristic: the testing sample usually locates at the region with fewer training samples. In other words, to use global or local information is less crucial for samples that are very close to existing samples. SVM is suitable for datasets with a good consistency among samples. For example, SVM performs well when using HU400 to predict HU216 in Table 5, because both datasets are extracted from the same species. RVKDE is suitable for datasets in which information is stored in local region, *i.e.*, to construct a global model for all the samples is not applicable. This echoes that RVKDE has some advantages when using human pre-miRNAs to predict pre-miRNAs from the genera taxonomically distant to humans.

In summary, SVM and RVKDE are two distinct classification mechanisms. SVM uses support vectors to model the global information of training samples and to prevent being misguided by a few noisy samples. RVKDE is instance-based and highly dependent on the local information of training samples. The variable variance of each kernel function (see the 'Materials and methods' section for details) makes RVKDE deliver better performance than conventional instance-based classifiers and achieve the same level of performance as SVM [35].

## Conclusion

There have been many efforts on discovering pre-miRNAs over the years. Recently, several *ab initio *approaches are especially of interest, because of the ability to discover species-specific pre-miRNAs that usually evaded by comparative approaches. This study develops a novel *ab initio *miRNA predictor by focusing on the classification mechanism. The adopted RVKDE exploits more local information from the training samples than widely used SVM. Experimental results show that the characteristic of exploiting more local information makes miR-KDE more suitable for species-specific miRNA prediction. The decision boundary analysis shows that alternative machine learning algorithms feature different advantages. These results encourage more efforts on the classification methodology as well as the feature extraction in miRNA prediction.

## Materials and methods

### Datasets

4039 miRNA precursors spanning across 45 species are downloaded from the miRBase registry database [39] (release 8.2). The CD-HIT clustering algorithm [40] with the similarity threshold set to 0.9 is then invoked to exclude homology sequences [25,28]. Pre-miRNAs whose secondary structures contain multiple loops are excluded. The resultant positive set contains 1983 non-redundant pre-miRNAs from 40 species, including 308 human pre-miRNAs.

For the negative set, we analyze 8494 pseudo hairpins from the protein-coding regions (CDSs) according to RefSeq [41] and UCSC refGene [42] annotations. These RNA sequences are extracted from genomic regions where no experimentally validated splicing event has been reported [25]. For each of the 8494 RNA sequences, we first predict its secondary structure by RNAfold [43]. RNA sequences with <18 base pairs on the stem, minimum free energy > -25 kcal/mol and multiple loops of the predicted secondary structure are removed. In summary, 3988 pseudo hairpins are collected. These pseudo hairpins are sequence segments similar to genuine pre-miRNAs in terms of length, stem-loop structure, and number of bulges but not have been reported as pre-miRNAs.

Based on the positive and negative sets, one training set and two test sets are built to evaluate the miRNA predictors. The training set, HU400, comprises 200 human pre-miRNAs and 200 pseudo hairpins randomly selected from the positive and negative sets, respectively. The HU400 dataset is used for parameter estimation and model construction of the miRNA predictors. The first test set, HU216, comprises the remaining 108 human pre-miRNAs and randomly selected 108 pseudo hairpins. The HU216 dataset is used to evaluate the prediction performance for human pre-miRNAs. Another test set, NH3350, comprises the remaining 1675 non-human pre-miRNAs and randomly selected 1675 pseudo hairpins. The NH3350 dataset is used to evaluate the prediction performance for species-specific pre-miRNAs. Table 6 shows a summary of these sets. Care has been taken to guarantee that no pseudo hairpin is included in the three datasets more than once.

**Table 6:** Summary of the datasets employed in this study.

**Dataset**	**Number of pre-miRNAs**	**Number of pseudo hairpins**	**Source of pre-miRNAs**
HU400	200	200	*Homo sapiens*
HU216	108	108	*Homo sapiens*
HU3350	1675	1675	39 non-human species

### Feature set

In miR-KDE, each hairpin-like sequence is summarized as a 33-dimensional feature vector. The first 29 features are derived from miPred [28], including 17 sequence composition variables, 6 folding measures, 1 topological descriptor, and 5 normalized variants. The 17 sequence composition variables comprises of 16 dinucleotide frequencies and the proportion of G and C in the RNA molecule. Other features including base pairing propensity [44], Minimum Free Energy (MFE) and its variants [45-47], base pair distance [46,48], Shannon entropy [46] and degree of compactness [49,50] have been shown useful in miRNA prediction.

**Figure 5:**
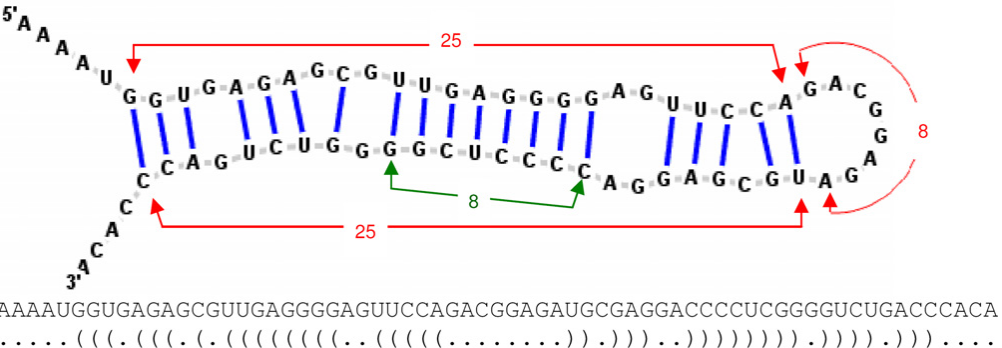
**The *Homo sapiens *miR-611 stem-loop structure**. The RNA sequence and its corresponding secondary structure sequence predicted by RNAfold [43] are shown. In the secondary structure sequence, each nucleotide has two states, "paired" or "unpaired", indicated by brackets and dots, respectively. A left bracket "(" indicates a paired nucleotide located at the 5' strand that would form a pair with another nucleotide at the 3' strand with a right bracket ")". The hairpin length of this sample pre-miRNA is 25+8+25 = 58. Its loop length is 8 and has 8 consecutive base pairs.

In addition, we introduce four additional features that focus on the continuously paired nucleotides on the stem and the loop length of hairpin structures. The four "stem-loop" features are based on the RNA secondary structures predicted with the RNAfold program [43]. Figure 5 shows an example of the predicted RNA secondary structure in which each nucleotide has two states, "paired" or "unpaired", indicated by brackets and dots, respectively. A left bracket "(" indicates a paired nucleotide located at the 5' strand that would form a pair with another nucleotide at the 3' strand with a right bracket ")". As shown in Figure 5, the first stem-loop feature is "hairpin length" defined as the number of nucleotides from the first paired nucleotide at the 5' strand to its partner, the last paired nucleotide at the 3' strand. The second stem-loop feature is "loop length" defined as the number of nucleotides between the last paired nucleotide at the 5' strand and its partner, the first paired nucleotide at the 3' strand. The third stem-loop feature is "consecutive base-pairs" defined as the number of longest successive base-pairs. The fourth stem-loop feature is the ratio of loop length to hairpin length.

### Relaxed variable kernel density estimator

MiR-KDE transforms samples into feature vectors as described above and then uses them to construct a relaxed variable kernel density estimator (RVKDE) [35]. A kernel density estimator is in fact an approximate probability density function. Let {**s**_1_, **s**_2 _...**s**_*n*_} be a set of sampling instances randomly and independently taken from the distribution governed by *f*_*X *_in the *m*-dimensional vector space. Then, with the RVKDE algorithm, the value of *f*_*X *_at point **v **is estimated as follows:

$\hat{f}(v)=\frac{1}{\left|n\right|}\sum_{s_{i}}{\left(\frac{1}{\sqrt{2\pi }\cdot \sigma_{i}}\right)}^{m}\exp\left(-\frac{||v-s_{i}|{|}^{2}}{2\sigma_{i}^{2}}\right),$, where

1) $\sigma_{i}=\beta \frac{R(s_{i})\sqrt{\pi }}{\sqrt[{m}]{\left(k+1)\Gamma (\frac{m}{2}+1\right)}}$;

2) *R*(**s**_*i*_) is the maximum distance between **s**_**i **_and its *ks *nearest training instances;

3) Γ (·) is the Gamma function [51];

4) *β *and *ks *are parameters to be set either through cross-validation or by the user.

For prediction of pre-miRNAs, two kernel density estimators are constructed to approximate the distribution of pre-miRNAs and pseudo hairpins in training set, respectively. As mentioned above, in our implementation, each RNA sequence is represented as a 33-dimensional feature vector. Then, a query instance located at **v **is predicted to the class that gives the maximum value among the likelihood functions defined as follows:

$$L_{j}(v)=\frac{\left|S_{j}|\cdot {\hat{f}}_{j}(v\right)}{\sum_{h}\left|S_{h}|\cdot {\hat{f}}_{h}(v\right)},$$

where |*S*_*j*_| is the number of class-*j *training instances, and ${\hat{f}}_{j}$(**v**) is the kernel density estimator corresponding to class-*j *training instances. In our current implementation, in order to improve the efficiency of the predictor, we include only a limited number, denoted by *kt*, of the nearest class-*j *training instances of **v **while computing ${\hat{f}}_{j}$(**v**). *kt *is also a parameter to be set either through cross-validation or by the user.

### Comparison between RVKDE and SVM

This subsection reveals some characteristics of RVKDE by comparing it to SVM. RVKDE belongs to the radial basis function network (RBFN), a special type of neural networks with several distinctive features [52,53]. The decision function of two-class RVKDE can be simplified as follows:

(1) $$f_{\text{RVKDE}}(v)=\sum_{s_{i}}y_{i}\cdot \frac{1}{\sigma_{i}}\cdot \exp\left(-\frac{||v-s_{i}|{|}^{2}}{2\sigma_{i}^{2}}\right),$$

where **v **is a testing sample. *y*_*i *_is the class value as either +1 (positive) or -1 (negative) of a training sample **s**_*i*_. *σ*_*i *_is the local density of the proximity of **s**_*i*_, estimated by the kernel density estimation algorithm. The testing sample **v **is classified as positive if *f*_RVKDE_(**v**) ≥ 0, and as negative otherwise. Interestingly, the decision function in Eq. (1) is very similar to the one in SVM using the radial basis function (RBF) kernel:

(2) $$f_{\text{SVM}}(v)=\sum_{s_{i}}y_{i}\cdot \alpha_{i}\cdot \exp(-\gamma ||v-s_{i}|{|}^{2}),$$

where *α*_*i *_(corresponds to $\sigma_{i}^{-1}$ in Eq. (1)) is determined by a constrained quadratic optimization [54] and *γ*(corresponds to 1/2$\sigma_{i}^{2}$ in Eq. (1)) is a user-specified parameter. According to Eq. (1) and (2), the mathematical models of RVKDE and SVM are analogous. The main difference between RVKDE and SVM is the criteria to determine *σ*_*i *_in Eq. (1) and *α*_*i *_in Eq. (2).

SVM uses support vectors to construct a special kind of linear model, maximum margin hyperplane, that separates the samples of different classes [54]. The *α*_*i *_in SVM is determined based on the global distribution of samples by maximizing the separation between the classes. Conversely, RVKDE uses only few samples (<10 in this study) in the proximity of a training instance and thus determines *σ*_*i *_based on local information. As the decision boundary plots reported in the 'Decision boundaries of SVM and RVKDE' subsection of this study, the effects of using global/local information are crucial in predicting pre-miRNAs.

### Experiment design

The proposed miR-KDE is evaluated by three experiments: 1) a five-fold cross-validation on the human pre-miRNA set HU400, 2) using the model trained by the first experiment to predict another human pre-miRNA set HU216 and 3) using the model trained by the first experiment to predict the non-human pre-miRNA set NH3350. Two SVM-based predictors, triplet-SVM and miPred, are included in these experiments for comparison. Parameters of alternative predictors are selected to maximize the accuracy of the first experiment. Five widely used indices for binary classification problems are introduced to evaluate the classifiers. Table 7 lists these performance measures.

**Table 7:** Evaluation measures employed in this study.

**Measure**	**Abbreviation**	**Equation**^1^
Sensitivity (recall)	%SE	TP/(TP+FN)
Specificity	%SP	TN/(TN+FP)
Accuracy	%ACC	(TP+TN)/(TP+TN+FP+FN)
F-measure	%Fm	2TP/(2TP+FP+FN)
Matthews' correlation coefficient	%MCC	(TP × TN-FP × FN)/sqrt((TP+FP) × (TN+FN) × (TP+FN) × (TN+FP))

^1^The definition of the abbreviations used: TP is the number of real pre-miRNAs detected; FN is the number of real pre-miRNAs missed; TN is the number of pseudo hairpins correctly classified; and FN is the number of pseudo hairpins incorrectly classified as pre-miRNA.

### Decision boundary plot

Before constructing a two-dimensional decision boundary plot, two features must be selected from the 29 features as the *x*-axis and *y*-axis. In this study, we want to identify the two features having most influence on the classification decision of the testing sample. A heuristic method is used to estimate the influence of each feature on the classification decision. According to Eq. (1) and Eq. (2), the classification decision is largely influenced by the nearest training samples to the testing sample, since the influence of a Gaussian function decreases exponentially as the distance increases. Furthermore, the distance ||**v **- **s**_*i*_|| in Eq (1) and Eq. (2) is more influenced by the dimensions with larger difference. Thus, the influence of a feature on the classification is estimated by the average of the differences of the testing sample to its *kt *nearest training samples (*kt *= 37 in this study). For each testing sample selected to generate a decision boundary plot, we estimate the influences of all 29 features. The feature with the most influence is selected as the *x*-axis, and the feature with the second most influence is selected as the *y*-axis.

In the decision boundary plots of this study, the black circle represents the testing sample, red circles represent the training pre-miRNAs and blue circles represent the training pseudo hairpins. The background color indicates the predictor's decision for a sample of which the two features equal to the *x*-axis and *y*-axis and the remaining 27 features equal to the testing sample. The boundary between red and blue background is the decision boundary of the classifier on the *xy*-plane. Notice that a blue circle over a red background, or vice versa, does not indicate that the predictor misclassifies that training sample. The training samples are projected onto this plane and have the remaining 27 features different to the samples represented by the background. Namely, these decision boundary plots show a slice near the testing sample of the vector space.

## Competing interests

The authors declare that they have no competing interests.

## Authors' contributions

Author DTHC participated in the development of RVKDE and conceived of this study. Both CCW and JWC designed the experiments and performed all calculations and analyses. All authors have read and approved this manuscript.
